# Rethinking Acute Sports Injuries: Evidence for an Overuse Mechanism in Hamstring and ACL Injuries

**DOI:** 10.1111/sms.70146

**Published:** 2025-10-13

**Authors:** Bas Van Hooren

**Affiliations:** ^1^ Department of Nutrition and Movement Sciences, NUTRIM School of Nutrition and Translational Research in Metabolism Maastricht University Medical Centre+ Maastricht the Netherlands

**Keywords:** ACL, acute, damage, hamstrings, injury, mechanical fatigue, modeling, overuse

## Abstract

Sports injuries have traditionally been classified as acute or overuse based on their onset and associated circumstances. Hamstring strain injuries and anterior cruciate ligament (ACL) injuries are two common sports injuries that are typically implicitly considered to represent acute injuries. This brief review, however, argues that hamstring and ACL injuries may at least partly present as overuse injuries resulting from a mechanical fatigue phenomenon, rather than acute injuries. Human, animal, and cadaveric studies are discussed to support this view. For example, human studies show no kinematic deviation in the stride during which the hamstring injury occurs as compared to the preceding strides. Further, the location of injury and ultrastructural damage of hamstring injuries is largely comparable to that seen in repetitive muscle–tendon unit lengthening experiments in animals. For the ACL, repetitive simulated jump landings have been shown to lead to ACL failure despite the ACL load being well below its ultimate strength. Furthermore, analyses of ACL explants obtained from noncontact ACL‐injured patients during reconstruction surgery indicate similar damage to cadaveric studies that repetitively loaded the ACL. In summary, studies with diverse methodological approaches support the view that mechanical fatigue may predispose hamstring and ACL tissues to failure at submaximal loads during seemingly normal movements. Although further research is needed to substantiate these hypotheses, recognizing mechanical fatigue as a factor in these injuries can inform training and rehabilitation protocols and open opportunities to use modeling approaches and wearable sensors to monitor tissue load and damage, ultimately reducing injury rates.

## 1 Introduction

**FIGURE 1:**
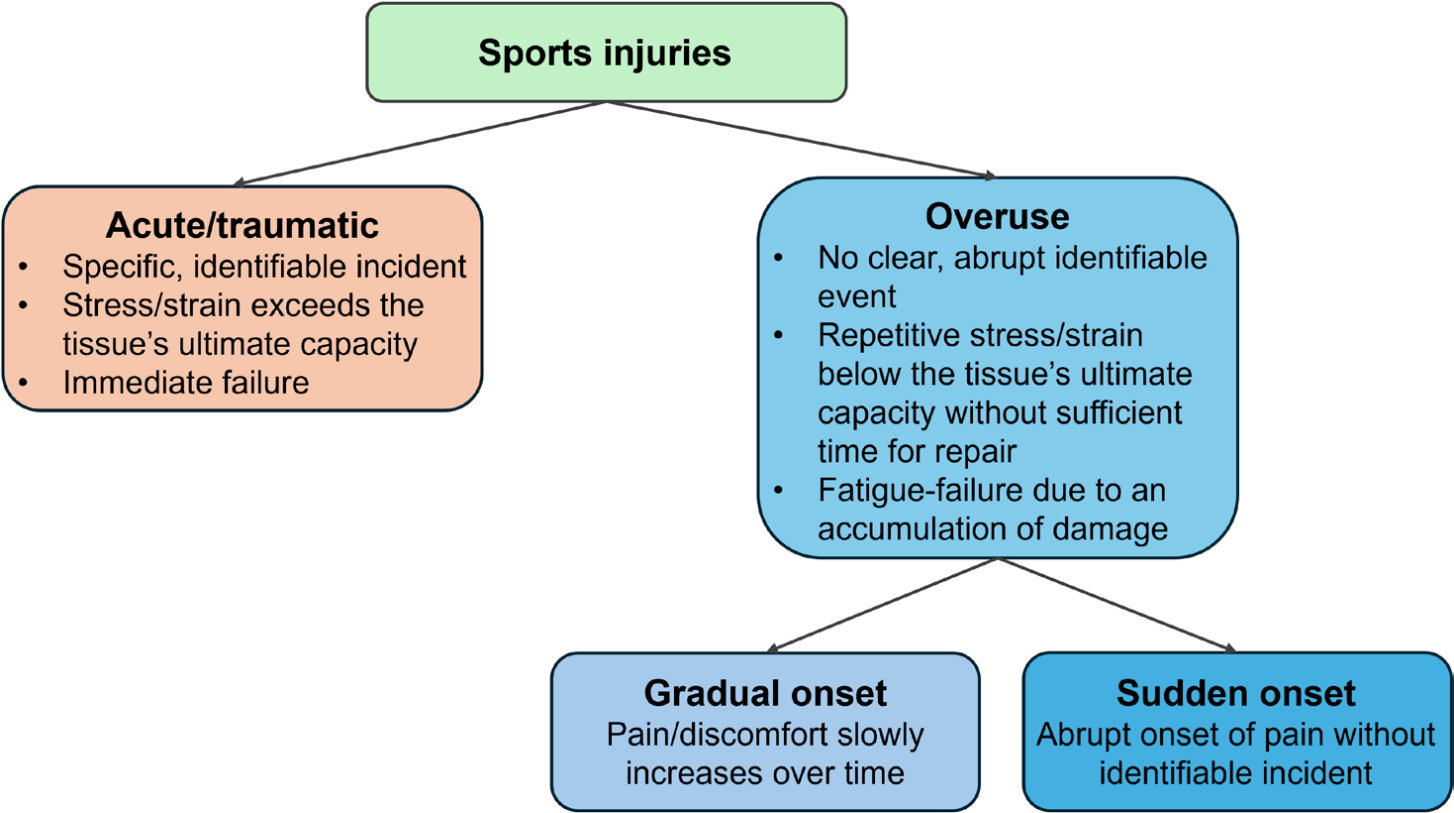
Schematic of acute and overuse injuries, with subcategories for gradual and sudden‐onset overuse injuries.

Sports injuries have traditionally been classified into acute/traumatic (sudden‐onset) and overuse (both gradual and sudden‐onset [1]) injuries [2, 3, 4, 5, 6]. An acute injury classification is typically made if the injury can be linked to a specific, identifiable incident, whereas an overuse injury classification is made if there is no clear, abrupt, identifiable event related to the injury (Figure 1). Notably, overuse injuries can further be distinguished based on whether pain slowly increases over time (e.g., from discomfort to pain) or whether pain suddenly appears, without a clear, abrupt, identifiable event (i.e., sudden‐onset overuse injury).

Two common sports injuries that are often (implicitly) considered to represent acute injuries include hamstring strain injuries [7, 8, 9] and anterior cruciate ligament (ACL) injuries [9]. The reason for this classification of these injuries is that there is often an identifiable incident related to the injury, such as a specific stride after which an athlete grabs for their hamstrings, or a specific change of direction after which an athlete reaches for their knee. Here, I argue that such injuries may at least partly present as overuse (sudden‐onset) injuries resulting from a mechanical fatigue phenomenon, rather than acute injuries. After presenting evidence to support this view, I also discuss some limitations of the current evidence with directions for future research, and implications of explicitly re‐considering these injuries as overuse injuries.

## 2 Acute Versus Overuse Injuries

**FIGURE 2:**
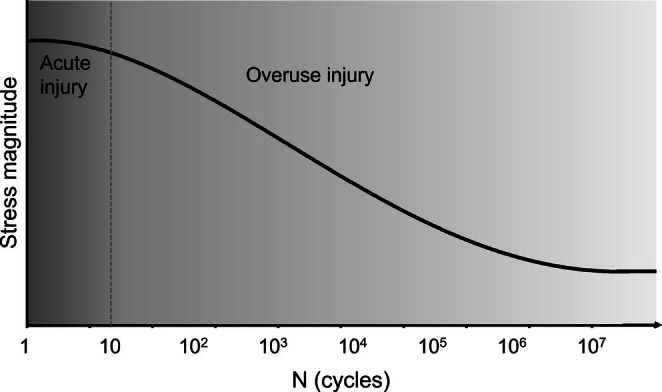
Schematic stress–life plot (S‐N curve) depicting the relationship between an applied stress and the number of cycles the tissue (bone, tendon, muscle, and cartilage) can endure before failing (i.e., the tissue's fatigue life). Importantly, tissues can fail due to a single stress or strain that exceeds the tissue's ultimate (tensile) strength (‘acute injury’), but can also fail with repetitive submaximal loading (‘overuse injury’). Note that the x‐axis is on a logarithmic scale. The implication of this logarithmic scale is that a small change in the magnitude of the stress has a large effect on the number of cycles the tissue can endure before failing.

From a mechanical perspective, acute injuries occur when a tissue is subjected to a stress or strain exceeding its ultimate stress or strain limit, leading to immediate structural or functional failure (e.g., rupture or fracture; Figure 2). Examples of acute injuries include bone fractures or ligament tears caused by high‐impact events such as falls from a large height or collisions at high speed. Hamstring injuries and ACL injuries have traditionally also implicitly been considered to represent acute injuries whereby the stress or strain during a movement exceeds the tissue's ultimate stress or strain limit.

Overuse injuries result from mechanical fatigue, where repetitive submaximal loading without sufficient time for repair degrades the material's strength such that failure can occur at stresses or strains well below the ultimate stress or strain limit (Figures 1 and 2) [10]. The resulting failure is often referred to as fatigue failure. Indeed, failure can first occur at the nanoscale (e.g., unraveling of the collagen molecular triple helix), and with repetitive loading, spread to the microscale (e.g., collagen fiber rupture), ultrastructural scale (e.g., partial tear), and even macroscopic scale (e.g., complete rupture). This mechanical fatigue phenomenon has been well established in both human cadaveric [11, 12, 13] and animal [14, 15, 16] experiments of various tissues, including bones [13, 14, 16], ligaments [15, 17], cartilage [11, 16], tendons [12, 16], muscles [16, 18, 19, 20], and nerves [16]. Examples of injuries that are attributed to mechanical fatigue (i.e., overuse injuries) include bone stress fractures and tendinopathy in sports such as distance running. In this context, it is important to note that while the strength of the tissue progressively weakens over time in overuse injuries, pain may manifest suddenly rather than gradually [1] (i.e., a sudden‐onset overuse injury; Figure 1). This may give the incorrect impression that the injury represents an acute injury instead of an overuse injury.

## 3 Hamstring Injuries

Hamstring injuries are among the most frequent injuries in football, which, combined with the moderate number of days lost per injury, leads to a relatively high injury burden [3]. The majority of hamstring injuries occur during high‐speed running and accelerations [9, 21, 22, 23], with the late swing phase being the most likely phase for injury occurrence [24, 25]. Although sprint‐type hamstring injuries are often considered acute injuries, emerging evidence suggests that these injuries may at least partly present as overuse injuries, rather than acute injuries.

**FIGURE 3:**
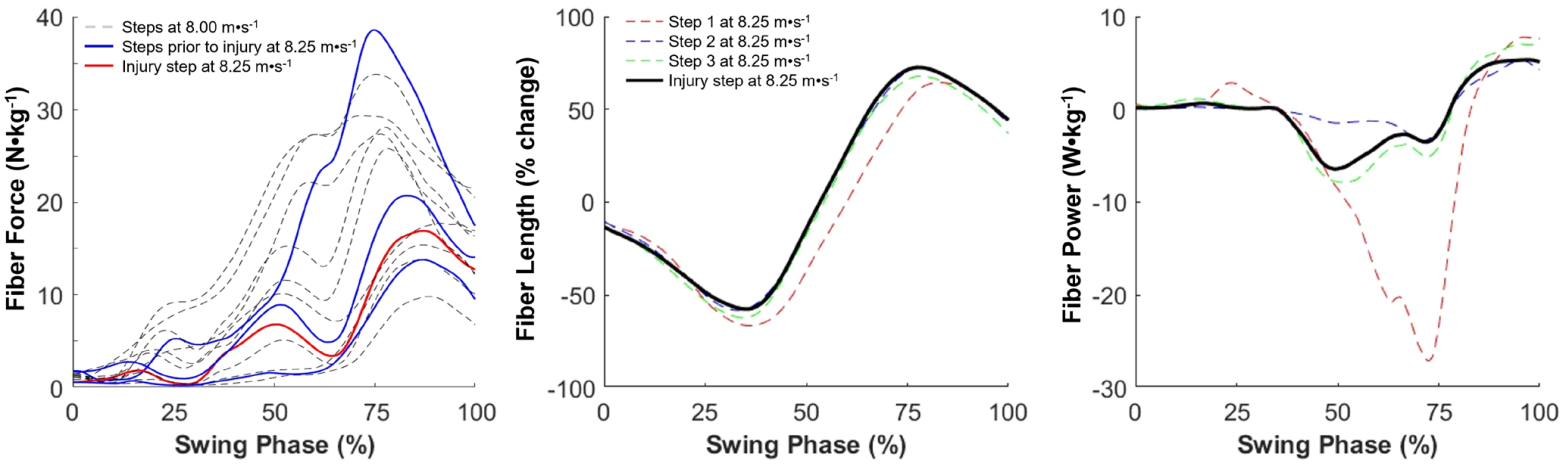
Biceps femoris relative muscle fiber force (left), length (middle) and power (right) in the three steps preceding a grade 2 biceps femoris injury, and the step with the injury. Note that the step wherein the injury occurred does not differ substantially from the preceding steps, thus hinting at a fatigue‐failure mechanism of hamstring injuries. For the panel with fiber forces, the fiber forces from a slightly slower speed (8.00 m·s^−1^) are also depicted to better indicate the variability in fiber force in a larger number of steps relative to the injury step. Adapted from [27]. Negative power values indicate energy absorption.

As discussed before, the occurrence of an acute injury requires that the time point at which the injury occurs is characterized by higher tissue loading than the tissue's ultimate stress or strain limit, and thus higher than routinely experienced by the tissue. Three case studies have captured running biomechanical data prior to, and during the occurrence of a hamstring injury, but did not find any notable kinematic or kinetic deviations in the stride during which the injury occurred compared to the preceding strides (Figure 3) [24, 26, 27]. This indicates no kinematic or kinetic reason for the injury occurrence during the specific stride, but instead suggests that the injury may have presented as an overuse injury resulting from an accumulation of nano‐ and microscopic damage, leading to an injury during a seemingly normal movement. In support of this, both modeling [28, 29] and ultrasound [30, 31] studies have shown the hamstrings to eccentrically lengthen during a part of the swing phase of high‐speed running, with the magnitude of length change (strain) being well below the ultimate strain limit [30]. Specifically, the hamstring operating length during late swing is estimated to be close to optimal length [24, 26, 27], which is well below the limit where muscles reach their ultimate strain, leading to a macroscopic tear (~12% for the biceps femoris long head under passive stretching [32]). Eccentric muscle actions are known to generate muscle damage [33], and the repetitive submaximal active lengthening, therefore, may have led to an accumulation of nano‐ and microscopic damage, which eventually resulted in a muscle (tendinous) tear (i.e., a hamstring injury) at the ultrastructural level [34]. In further support of this, hamstring injuries have also been shown to occur at submaximal running speeds [7], which would be expected to place the hamstrings even further from their ultimate strain and stress limit [30, 31]. Furthermore, individuals with a hamstring injury have been found to exhibit higher workload values in the week preceding the injury when compared to the preceding 6 weeks [35], consistent with the overuse hypothesis.

The hypothesis that a part of the hamstring strain injuries represent overuse injuries is further supported by comparing the nature of the damage from hamstring injuries to studies that have induced fatigue‐failure (overuse) injuries to muscle–tendon units in cadaveric humans or animals. The ultrastructural damage occurring in lab‐based fatigue‐failure studies (e.g., edema) [36] is, for example, comparable to that seen with magnetic resonance imaging in humans with hamstring injuries [37]. Further, repetitive active or passive lengthening of a muscle–tendon unit typically results in failure at the myotendinous junction [36]. This failure location is similar to that of hamstring injuries, where most injuries also involve the myotendinous junction [37]. It is important to note, however, that lab‐based studies often also report this failure to occur at the distal myotendinous junction, whereas hamstring injuries often (but not exclusively) occur at the proximal myotendinous junction [37]. Differences in the assessed muscle–tendon units (e.g., typically calf muscles) and exact loading protocols may explain this discrepancy. Interestingly, a single passive elongation of the human hamstrings in cadavers until failure shows rupture to occur at the proximal myotendinous junction [32]. While this may be interpreted as evidence that in vivo hamstring injuries are likely acute injuries because they also typically involve the proximal myotendinous junction, failure may occur likewise at the proximal location with repetitive loading. In support of this, animal studies of the calf muscles show both repetitive [36] and single [38] loading experiments typically involve the same (distal) myotendinous junction, suggesting the human hamstrings may also fail at the proximal myotendinous junction with repetitive loading.

## 4 ACL Injuries

Although ACL injuries occur less frequently than hamstring injuries, they lead to a substantially higher number of days lost, which therefore also leads to a high overall injury burden [3]. Noncontact ACL injuries represent the majority of ACL injuries [39] and occur primarily during abrupt deceleration, landing, and pivoting maneuvers [40, 41, 42]. It is thought that these movements lead to excessive strain/stress of the ACL, which in turn leads to an acute injury (i.e., ACL tear). However, abrupt decelerations, landings, and pivoting maneuvers are routinely performed without ACL injuries. For example, Premier League soccer players perform on average more than 700 changes of direction during a match [43]. Moreover, a substantial proportion of ACL injuries also involve activities with moderate horizontal speeds as opposed to higher speeds [39]. These observations suggest that ACL injuries may also at least partly present overuse injuries whereby nano‐ and microdamage accumulates over many loading cycles [44, 45], thereby leading to failure (i.e., mechanical fatigue) during one seemingly normal movement. While this hypothesis has been proposed before [44], the evidence and the implications were discussed only briefly. As a result, this view has not been broadly taken up by the sport science and sport medicine community, with recent studies still largely referring to an acute injury mechanism, thus necessitating a more elaborate discussion of the evidence for an overuse mechanism in ACL injuries.

**FIGURE 4:**
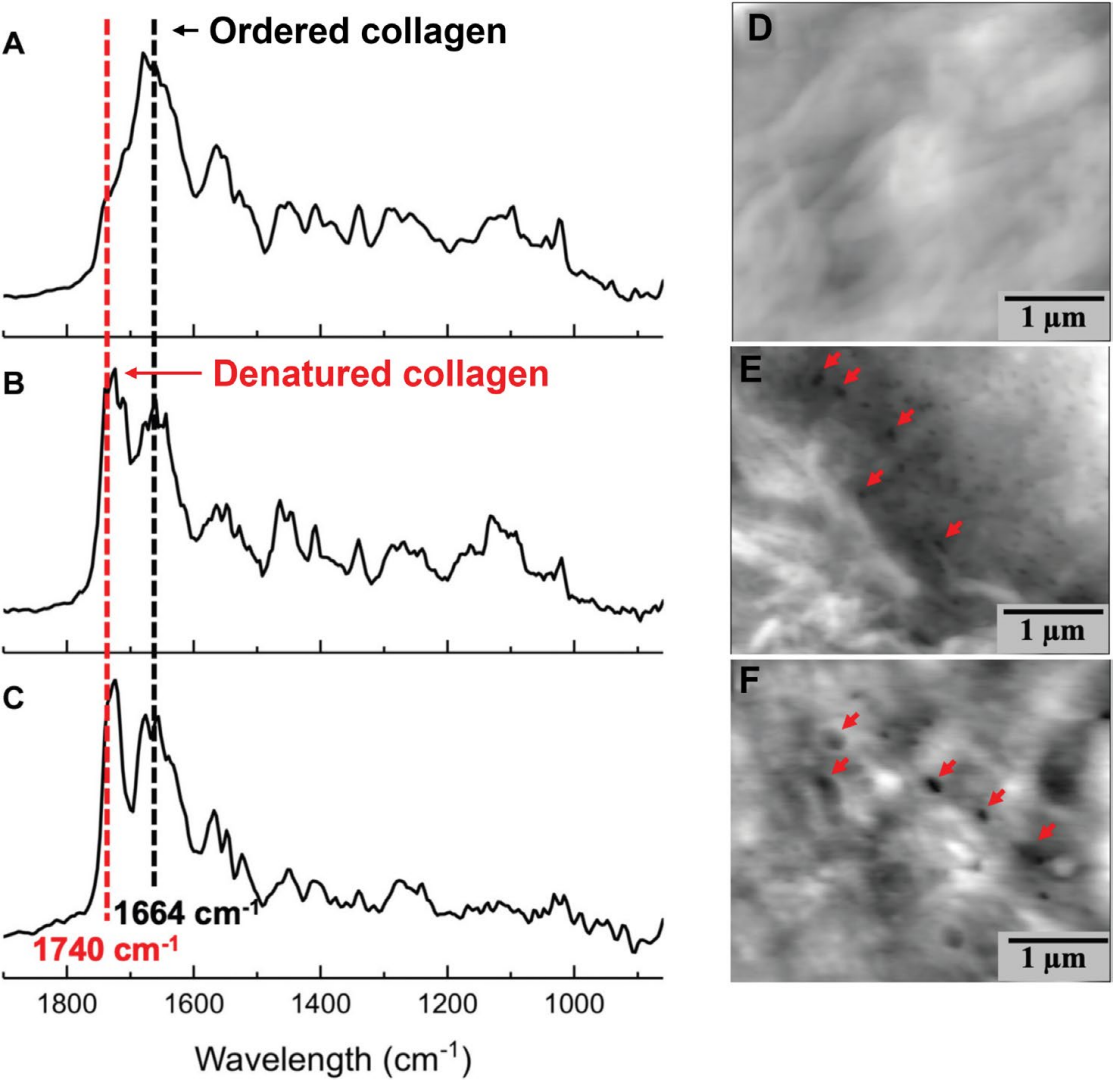
Example nanoscopic damage in an ACL patient and ACL cadaveric specimen after repeated mechanical loading. Left: Atomic force microscopy–infrared spectroscopy spectra of tissues at the anterior cruciate ligament (ACL) femoral enthesis from (A) an apparently healthy (nonloaded) cadaveric ACL, (B) a cadaveric ACL that has undergone repeated mechanical loading, and (C) a patient with ACL injury at the time of surgical reconstruction. Note that the cadaveric ACL tissue (B) and patient‐derived ACL tissue (C) show a very similar increase in the 1740 cm^−1^ spectrum (red dashed line; representing denatured/disorganized collagen), whereas the control ACL tissue shows a predominant band in the 1664 cm^−1^ spectrum (black dashed line; representing ordered collagen). Right: Atomic force microscopy images of fibril voids in the anterior cruciate ligament (ACL). (D) depicts a cadaveric ACL femoral enthesis, showing no presence of locations without collagen molecules. In contrast, both the cadaveric ACL femoral enthesis after repeated mechanical loading (E) and the explant from a patient with ACL injury at the time of surgical reconstruction (F) show locations without collagen molecules (darker contrast as indicated by the red arrows). Adapted with permission from [46].

Several lines of evidence support this view. First, cadaveric and animal studies have shown that repetitive ACL loading leads to nano‐ and microscopic damage accumulation, such as collagen unraveling and voids [15, 17, 46, 47]. This damage accumulation weakens the ACL such that it fails at submaximal loads. Indeed, a cadaveric study showed that repetitive simulated jump landings led to ACL failure, despite the ACL load being well below the ultimate strength limit [48]. Although cadaveric studies have several limitations (e.g., typically older individuals and challenges in mimicking muscle activation and forces), analyses of ACL explants obtained from noncontact ACL‐injured patients during reconstruction surgery indicated similar damage signs to cadaveric studies that repetitively loaded the ACL (Figure 4) [46]. This further reinforces the idea that submaximal loading may reduce structural integrity and lead to overuse injury when there is insufficient time for tissue repair. More support for the idea that the ACL can fail due to an accumulation of microdamage comes from prospective and retrospective studies in humans. A retrospective cohort study, for instance, reported that athletic activities and maneuvers deemed “risky” in relation to ACL injuries increased in the 6‐month period prior to sustaining an ACL injury [49]. Furthermore, ACL T_2_ relaxation times increased during the competitive season in three professional Alpine skiers [50]. Although cause and effect cannot be determined from this study design, the longer T_2_ relaxation times may reflect lower cell density and collagen organization, as well as lower tensile load‐bearing capacity before failure. Similarly, ACL cross‐sectional area has been reported to increase during a soccer season [51], with this increase possibly reflecting inflammation and remodeling in response to accumulated microtrauma. Finally, ACL tears have been observed even in the absence of traumatic events [52], hinting at an overuse mechanism.

## 5 Limitations of Current Evidence and Recommendations for Future Research

While several lines of evidence support the hypothesis that at least a part of hamstrings and ACL injuries may represent overuse injuries, there are several limitations to the present body of evidence that should be considered.

First, all case studies done thus far on hamstring injuries have estimated whole‐muscle forces and strains. Localized stresses and strains could therefore still exceed the local ultimate limit and thereby differ from whole muscle outcomes [53], for example, due to different structural properties or altered neural activation (e.g., fatigue‐related cramp). Future studies may explore the effects of altered localized neural activation using modeling or cadaveric and animal experiments to assess whether this may lead to acute injuries during repetitive loading experiments. Furthermore, the case studies on hamstring injuries also used generic muscle–tendon parameters, and tissue stresses and strains may further differ depending on individual morphological differences. Future research should therefore attempt to individualize models used for case studies to ensure a better estimation of tissue loading. Second, most muscle–tendon unit fatigue‐failure studies have used the calf muscles of animals, and the generalizability to the human hamstrings is unknown.

With regard to the ACL, it should be considered that fatigue‐failure experiments using cadaveric specimens have typically used middle‐aged individuals, whereas ACL injuries typically occur in younger individuals in vivo. The use of middle or older‐aged cadaveric specimens in fatigue‐failure experiments may, however, be considered a strength in the context of overuse injuries. Specifically, increases in cadaveric age are associated with decreases in the ligament's ultimate strength [54] and the absence of acute failure in loading protocols mimicking high‐intensity sports activities, even in these aged specimens, therefore, support the notion that ACL injuries in vivo may involve cumulative microtrauma and overuse, rather than being solely the result of a single, catastrophic event. Similarly, while most studies report no significant changes to muscle or ligament ultimate strengths within up to 48 h post mortem [55] or after freezing [56], the absence of acute tissue failure despite the possible decreases in tissue integrity that occur post mortem further strengthens an overuse hypothesis. A limitation to ACL‐loading cadaveric studies is, however, that it is challenging to accurately mimic muscle activation, in particular of unplanned movements, or during fatigue. It could be hypothesized that the ACL loads may remain below the ultimate strain limit during planned and nonfatigued conditions (e.g., as simulated in cadavers by [48]) when, for example, the hamstrings co‐contract adequately [57]. However, further research is required to estimate if the stress and strain also remain below the ultimate limit during nonplanned changes in direction and during fatigued conditions, where muscle activation may be altered such that it increases the stress and strain on the ACL.

To overcome the limitations of the present evidence in relation to an overuse mechanism for hamstring and ACL injuries, future studies may combine human cadaveric hamstring or ACL loading experiments with prospective high‐volume sprint or ACL‐straining activities. This design, for example, allows comparison of the damage generated by repetitive human cadaveric hamstring loading to the damage seen in vivo with imaging methods, or to the damage observed in muscle samples obtained from athletes that sustain a noncontact hamstring injury and require surgery. The benefit of the latter approach is that it allows for the use of higher resolution imaging methods because most noninvasive imaging methods have limited resolution to detect nano‐ and microscopic damage. Nevertheless, there are several imaging methods worthwhile applying in future research (e.g., [58, 59, 60, 61]). The benefit of imaging methods is that they allow tracking of damage accumulation over time, as opposed to one time point only, with the use of surgical specimens. There are a few additional important considerations for such a repeated‐measures study design. First, cadaveric reference fatigue‐failure studies need to accurately mimic the forces and neuromuscular activation of the hamstrings to ensure the findings replicate in vivo hamstrings functioning. Furthermore, they should ideally use cadaveric samples representative of young adults and use tissue preparation methods that best replicate in vivo tissue stiffness. A first step would therefore involve validation of the cadaveric loading protocol for accurately representing in vivo loading. The high‐volume sprint or ACL‐straining activities protocol should be performed over several weeks, and include relatively short recovery between sessions to accumulate sufficient microdamage without repair. Furthermore, tissue loading should also be assessed in both cadaveric and in vivo studies, for example, using wearable sensors (see Section 6), to allow assessment of relationships between tissue loading (as opposed to loading inferred from, e.g., distance run) and tissue damage. A wearable tool that is broadly validated for estimating hamstring or ACL loading and damage still needs to be developed, but progress towards this has already been made [62, 63].

## 6 Implications

There are several implications of explicitly reconsidering hamstring and ACL injuries as overuse injuries. First, if hamstring and ACL injuries are considered as acute injuries resulting from a single event whereby the load exceeds the tissue capacity, a monitoring strategy is unlikely to be useful, as these events are arguably largely unpredictable. In contrast, when these injuries are considered to represent overuse injuries resulting from a mechanical fatigue phenomenon, monitoring of tissue load and the resulting damage, and provision of sufficient time for tissue repair after a given loading bout can both be useful to reduce injury risk. Such a strategy is well‐accepted in other overuse injuries. For example, it has long been established that ulnar collateral ligament injuries in baseball pitchers can result from repetitive loading of the ligament due to throwing at high velocities with insufficient recovery [64]. For this reason, guidelines have been implemented regarding the volume and frequency of pitches in baseball for youth and adolescent players [65]. Similar guidelines may be established for the number of high‐speed runs and accelerations in relation to hamstring injury risk, or the number of ACL‐straining activities such as high‐speed changes of direction. A drawback of such an approach is, however, that the load on the hamstrings or ACL is inferred from activities, and this may not accurately present the actual tissue load due to variability in movement execution, tissue geometry, and tissue mechanical properties. For example, the exact way by which a change of direction is performed can substantially influence ACL loading [44, 66], and differences in hamstring fascicle length or aponeurosis geometry [67] can also influence the damage resulting from a specific acceleration. Recent advancements in wearable technology may partly overcome these issues as wearables allow for monitoring of (personalized) tissue‐level loading during various in‐field activities. Recent studies have, for instance, used pressure insoles to quantify tissue‐level loading at common running injury locations during various running conditions [68, 69]. A similar approach may be used to estimate hamstrings and ACL load. Indeed, attempts have already been made to quantify tissue loading for the ACL from inertial measurement units in cadaveric experiments [70] and during a combined landing–cutting movement in humans [62]. Importantly, wearable‐based models need to consider the nonlinear relationship between load and damage [10, 71, 72], creep [62], and incorporate information on the recovery duration required after a given loading bout to accurately inform on injury risk and mitigation strategies. While all three aspects have been incorporated in a recent study aiming to predict ACL damage [62], to date, little knowledge is available on the load–damage relationship for (isolated) muscle and ACL tissue, or the recovery duration after a given cumulated load/damage for muscle and ligaments (e.g., [73]). Therefore, estimates from tendon load–damage relationships and tendon recovery times have been used instead [62], which reduces the accuracy of the model's estimates. Further research is therefore recommended in this area. Similarly, accurate information regarding damage and recovery duration from wearable sensors requires personalized models that take into consideration metabolic and structural factors. For example, individuals with chronic inflammation (e.g., diabetes patients) are known to have a higher risk of sustaining tendon and ligament injuries due to impaired tissue repair [74, 75]. Furthermore, nontraumatic ACL tears are more likely to occur in individuals with structural anomalies [52]. Moreover, females are also at higher risk of sustaining ACL injuries, among others, due to structurally weaker ligaments [76] and due to a larger increase in knee laxity [77], which in turn increases ACL strain and damage. Therefore, these additional factors also need to be considered when attempting to identify individuals at higher risk of sustaining an overuse injury. For example, baseline knee laxity levels derived from relatively easy‐to‐perform tests may be entered in a wearable application that predicts tissue‐level loading to better personalize the injury risk assessments [77, 78, 79]. Similarly, information is required on the strength of the tissue. While this requires methods that are not available for routine use at present [62, 80], in the future, such information may be derived from simple imaging methods available at local clinical practices.

Second, considering ACL and hamstring injuries as overuse injuries may also help to better contextualize conflicting findings regarding the effect of fatigue on hamstring and ACL injury risk. For example, studies report increased risk of hamstring injuries toward the end of each match half in soccer [81], thereby implying a role for fatigue, while most ACL injuries occur during the first half of a match [82, 83], suggesting a smaller role of fatigue. From an overuse injury perspective, this may be explained by fatigue both reducing and increasing overuse injury risk. Specifically, fatigue will reduce peak sprint speeds, accelerations, and jump heights, thereby reducing hamstrings and ACL loading and damage, while at the same time also altering kinematics and neural control in such a way that these structures experience higher loading [84, 85].

Third, increasing the tissue capacity to better tolerate repeated submaximal loading may be a viable strategy to reduce the risk of injuries. For example, increasing hamstring fascicle length may reduce the magnitude and velocity of active fiber lengthening [86], thereby reducing damage [87, 88]. Indeed, studies have reported that longer hamstring fascicle lengths reduce the risk of sustaining a hamstring injury [89]. Similarly, improving the ability of the muscle to remain quasi‐isometric during muscle–tendon unit lengthening may reduce the muscle fiber lengthening magnitude and velocity [90, 91, 92], thereby reducing damage. For the ACL, increasing its stiffness may reduce the length change for a given loading magnitude, thereby reducing damage. Similarly, a larger ACL cross‐sectional area due to training with sufficient recovery [93] will reduce the stress and thereby reduce injury risk (also for traumatic injuries). Regular physical activity (as opposed to, for example, once a week highly concentrated loading), sleep, and nutritional intake (e.g., collagen) may also facilitate tissue adaptation and recovery and are therefore also key aspects to reduce overuse injury risk [94].

Fourth, one aspect that characterizes overuse injuries is that the damage accumulates from the nanoscopic to the macroscopic level. Imaging methods that are currently used to assess tissue damage and recovery are, however, typically unable to detect nano‐ and microscopic level damage, and instead can only detect damage at larger scales (i.e., the ultrastructural level). For example, most magnetic resonance scanners and ultrasound systems have an in‐plane spatial resolution of approximately 0.3 mm–0.6 mm [95] and 0.06 mm–0.6 mm (assuming a 17–8 MHz linear transducer), respectively. However, the exact resolution depends on the method and scanner used, with higher resolutions available [61, 96]. Nevertheless, the typical spatial resolution significantly reduces their sensitivity for assessing the relationship between tissue loading and tissue damage, and means that only larger damage magnitudes, possibly resulting from a large single bout of exercise (e.g., marathon [61]) or from multiple training sessions, may be detected. However, tissue recovery already occurs between multiple training sessions, thus impairing the ability to determine relationships between tissue loading and damage in vivo humans. Such a challenge may be overcome by using cadaveric experiments, where tissue loading can be directly measured, and where nano‐ and microscopic damage may be assessed using invasive methods. A drawback of such an approach is, however, that this does not allow study of the relationship between tissue damage and tissue recovery, as repair is absent in cadaveric studies. Furthermore, cadaveric specimens do not often reflect healthy young athletes and may therefore not generalize well to the target population. A similar limitation is present in animal experiments. A combination of multiple approaches is therefore required to further our understanding of the relationship between tissue loading, tissue damage, and tissue repair time.

Finally, although this review has provided evidence for an overuse mechanism in hamstring and ACL injuries, a similar view may apply to other common injuries (e.g., calf, rectus femoris, and rotator cuff muscle tears, and injuries to other ligaments and tendons), and the discussed implications may therefore also prove useful to other overuse injuries. For example, soleus muscle injuries typically also involve the myotendinous junction [97], similar to repetitive active or passive calf muscle lengthening in animals [36]. Furthermore, rotator cuff tendinopathy is also characterized by collagen fragmentation and disorganization [98], similar to what is seen in repetitive tendon loading experiments. Nevertheless, in vivo studies with high‐resolution methods are required to further validate the overuse hypothesis for all these tissues (including the hamstrings and ACL).

## 7 Conclusion

Hamstring strain and ACL injuries may at least partly present as overuse injuries instead of acute injuries. Explicitly reconsidering these injuries from this perspective opens opportunities for monitoring tissue‐level loading with wearable technology and the combination of the derived load and damage with remodeling processes. Finally, viewing these injuries as overuse injuries may also shed a different light on injury prevention strategies.

## Conflicts of Interest

The author declares no conflicts of interest.

## Data Availability

Data sharing not applicable to this article as no datasets were generated or analysed during the current study.
